# Immune Checkpoints in Viral Infections

**DOI:** 10.3390/v12091051

**Published:** 2020-09-21

**Authors:** Huiming Cai, Ge Liu, Jianfeng Zhong, Kai Zheng, Haitao Xiao, Chenyang Li, Xun Song, Ying Li, Chenshu Xu, Haiqiang Wu, Zhendan He, Qinchang Zhu

**Affiliations:** 1 Guangdong Key Laboratory for Genome Stability & Human Disease Prevention, School of Pharmaceutical Sciences, Shenzhen University, Shenzhen 518060, China; 2014224055@email.szu.edu.cn (H.C.); lggege@szu.edu.cn (G.L.); 2016224006@email.szu.edu.cn (J.Z.); zhengk@szu.edu.cn (K.Z.); xhaitao@szu.edu.cn (H.X.); lcy@szu.edu.cn (C.L.); xsong@szu.edu.cn (X.S.); li.ying@szu.edu.cn (Y.L.); cshuxu@szu.edu.cn (C.X.); wuhq@szu.edu.cn (H.W.); 2 Shenzhen Key Laboratory of Novel Natural Health Care Products, Innovation Platform for Natural Small Molecule Drugs, Engineering Laboratory of Shenzhen Natural Small Molecule Innovative Drugs, Shenzhen University Health Science Center, Shenzhen 518060, China

**Keywords:** immune checkpoint, virus, chronic infection, immunotherapy

## Abstract

As evidence has mounted that virus-infected cells, such as cancer cells, negatively regulate the function of T-cells via immune checkpoints, it has become increasingly clear that viral infections similarly exploit immune checkpoints as an immune system escape mechanism. Although immune checkpoint therapy has been successfully used in cancer treatment, numerous studies have suggested that such therapy may also be highly relevant for treating viral infection, especially chronic viral infections. However, it has not yet been applied in this manner. Here, we reviewed recent findings regarding immune checkpoints in viral infections, including COVID-19, and discussed the role of immune checkpoints in different viral infections, as well as the potential for applying immune checkpoint blockades as antiviral therapy.

## 1. Introduction

Viral infections, especially chronic viral infections, are still a major threat to global health. Currently, the world is facing the serious challenge of a viral pandemic (coronavirus 2019; COVID-19) caused by severe acute respiratory syndrome coronavirus 2 (SARS-CoV-2), which has infected over 28,000,000 people and resulted in nearly 930,000 deaths globally. Additionally, more than 325 million people worldwide are infected with chronic hepatitis B [1] and about 1.2 million people worldwide die each year from acquired immune deficiency syndrome (AIDS) and related diseases [2]. In chronic viral infections, the virus escapes elimination by the immune system and establishes a persistent infection by modulating or regulating the host immune response [3]. Many chronic viral infections result in T-cell exhaustion, which is the main source of host difficulty in eliminating such infections [3,4]. As a negative regulatory signal for the activation and proliferation of T-cells, the immune checkpoint pathway is involved in the immune escape of many viruses [5,6].

Immune checkpoint molecules are negative regulatory receptors expressed on immune cells. Under normal physiological conditions, they function as a brake for the immune system, maintaining self-tolerance and preventing immunopathology in the body [7]. However, these molecules have also been shown to participate in the mechanism of immune escape by causing T-cell dysfunction in a variety of diseases, such as cancer and infection. The expression of immune checkpoint molecules on suppressor cells, such as regulatory T- (Treg) and regulatory B (Breg)-cells, could affect the function and cytokine secretion of these cells. Although the concept of immune checkpoints was first proposed in 2006 [8], research on the checkpoint receptors began much earlier. Allison discovered cytotoxic T-lymphocyte-associated protein 4 (CTLA-4) in 1995 and began studying the therapeutic effect of anti-CTLA-4 antibody on tumors [9,10], and Honjo discovered programmed death-1 (PD-1) in 1992 [11]. Since then, additional immune checkpoint molecules, such as T-cell immunoglobulin and mucin domain-containing-3 (TIM-3) and lymphocyte activation gene-3 (LAG-3), have been discovered [12,13]. To date, at least six immune checkpoints have been found to be involved in viral infections.

Conventional antiviral therapy is usually incapable of eliminating chronic infection [14]. However, recent advances in cancer immunotherapy may be applicable as antiviral therapy for chronic viral infections. Seven immune checkpoint inhibitors (ICIs) targeting CTLA-4, PD-1, or programmed death-ligand-1 (PD-L1) have been approved for the treatment of certain cancers and have shown positive therapeutic outcomes in patients [15,16]. Moreover, as a new approach for effective T-cell activation, combination therapy targeting multiple immune checkpoints or applied with other therapeutic modalities such as vaccines are currently being tested in clinical trials [17]. Here, we reviewed the recent findings regarding immune checkpoints in viral infection. We also discussed the role of immune checkpoints in different viral infections and the potential of applying immune checkpoint blockades as antiviral therapy.

## 2. Immune Checkpoints and Their T-cell Inactivation Pathways

**Figure 1:**
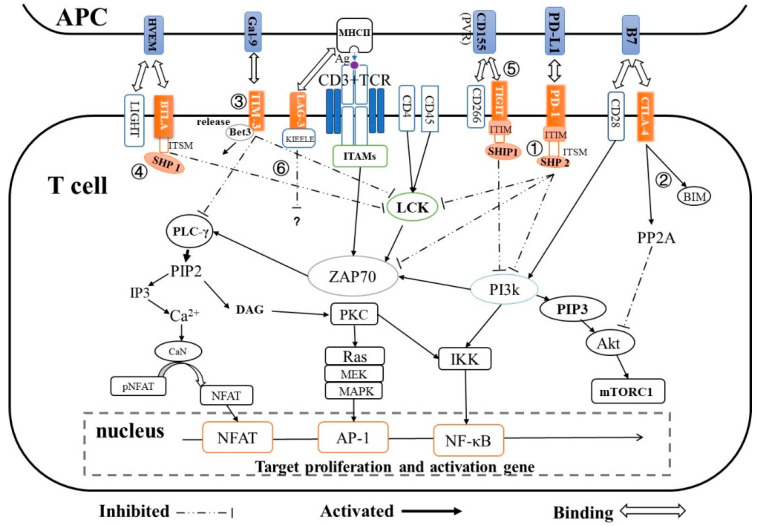
Mechanism of immune checkpoint-mediated T-cell inactivation. ①: PD-1/PD-L1 inhibits the PI3K/AKT pathway or ZAP70 phosphorylation by recruiting SHP2 phosphatase; ②: CTLA-4 competitively binds to the B7 ligand of CD28 and directly inhibits Akt by activating the phosphatase PP2A, and induces proapoptotic protein BIM; ③: TIM-3/Gal-9 releases Bat3, the molecule that binds to the intracellular tail of Tim-3, which allows Tim3 to bind to Lck or PLC-γ, leading to NF-κB and NFAT inhibition; ④: BTLA/HVEM recruits SHP-1, leading to the inhibition of LCK-dependent T-cell activation; ⑤: TIGIT/CD155 directly inhibits T-cell activation and proliferation by countering the costimulatory function of CD226, and also inhibits PI3K and MAPK signaling pathway by recruiting SHIP-1; ⑥: Lag-3 downregulates T-cell activation through a still unclear mechanism. Abbreviations: ITAMs, immunoreceptor tyrosine-based activation motif l; LCK, lymphocyte-specific protein tyrosine kinase; ZAP70, zeta chain of T-cell receptor associated protein kinase 70; PLC-γ, Phospholipase C-γ; PI3K, phosphatidylinositol 3-kinase; PIP3, phosphatidylinositol (3,4,5)-trisphosphate; PIP2, phosphatidyl inositol(4,5) bisphosphate; IP3, inositol-1,4,5-trisphosphate; DAG, diacylglycerol; PKC, protein kinase C; CaN, Calcineurin; IKK, inhibitor of nuclear factor kappa-B kinase; Akt, protein kinase B (Also known as PKB or Rac); PP2A, Protein phosphatase 2 A; Ras/MEK/MAPK, Ras GTPase-protein/MAP kinase kinase/MAP kinase pathway; mTORC1, mammalian target of rapamycin complex 1; NFAT, nuclear factor of activated T-cells; pNFAT, phospho NFAT; AP-1, activator protein 1; NF-κB, nuclear factor-κB.

The immune checkpoint coinhibitory network acts by inhibiting T-cell activation through various mechanisms and signaling pathways (Figure 1, Table 1).

PD-1 is preferentially expressed on the surface of activated T-cells and B-cells. Its expression has also been observed on the surface of other immunocyte subsets, such as natural killer (NK) cells, monocytes, and dendritic cells (DCs) [18,19]. The cytoplasmic tail of PD-1 has an immunoreceptor tyrosine-based inhibition motif (ITIM) and an immunoreceptor tyrosine-based switch motif (ITSM). When it binds to its ligand PD-L1/PD-L2, PD-1 translocates to T-cell receptor (TCR) microclusters and recruits SH2 domain-containing tyrosine phosphatase 2 (SHP2) by phosphorylating tyrosine residues in the ITSM [20]. Subsequently, the dephosphorylation of Zap-70 and LCK by SHP2 leads to inhibition of the Ras–MEK–MAPK pathway and interference with CD28 costimulatory signaling [21,22]. Additionally, SHP2 recruitment blocks the activation of phosphoinositide 3-kinase (PI3K) by completely binding to the phosphorylation kinases, then inhibits downstream Akt activation, which ultimately downregulates the activation of T-cells. A recent study suggested that the immune inhibitory function of PD-1 may occur via an SHP2-independent pathway [23]. PD-1 also can inactivate T-cells by promoting microcluster formation and degrading TCR on the surface of T-cells [22].

CTLA-4 is an important co-inhibitory receptor on T-cells that downregulates T-cell activation and induces tolerance [24]. It is transiently expressed following T-cell activation and blocks the costimulatory effect of constitutively expressed CD28 by competing with CD28 for CD80 binding [25]; it then inhibits Akt activation by recruiting PP2A [26,27]. Based on work in murine T-cells, CTLA-4 engagement results in T-cell suppression by limiting nuclear factor (NF)-κB and AP-1 transcriptional activity [28,29]. Recent research found that CTLA-4 expression induces high levels of the proapoptotic protein Bim in CD8^+^ T-cells and promotes Bim-dependent apoptosis in T-cells [30].

TIM-3 is selectively expressed on CD4^+^ T helper (Th)1 or Th17 cells [31], CD8^+^ T cytotoxic 1 (Tc1) cells, and Treg cells, and this expression can lead to inhibition of the Th1 response and apoptosis of antigen-specific cells. Under normal circumstances, the molecular adaptor human leukocyte antigen B (HLA-B)-associated transcript 3 (Bat3), which binds to the intracellular tail of TIM-3, protects Th1 cells from TIM-3-mediated cell death or exhaustion. When galectin-9 (Gal-9) binds to TIM-3, Bat3 is released from TIM-3, allowing TIM-3 to bind to the SH3 domain-containing TCR-associated intracellular kinase LCK and then to mediate downregulatory signals that inhibit Th1 responses [32]. Some research has suggested that TIM-3 suppresses T-cell activation by suppressing the nuclear factor of activated T-cells (NFAT) dephosphorylation and AP-1 transcription [33,34].

T-cell immunoglobulin and ITIM domain (TIGIT) is typically expressed on activated human T-cells, human NK cells, memory T-cells, and Treg cells. When TIGIT binds to CD155 (PVR) or CD112 (PVRL2 or Necl5) expressed on antigen-presenting cells (APCs), an inhibitory signal for T-cell activation is directly transmitted via the cytoplasmic tail of TIGIT [35]. The phosphorylated ITT-like motif in the TIGIT cytoplasmic tail binds the cytosolic adapter growth factor receptor bound protein 2 (Grb2) and then recruits SH2-containing inositol phosphatase-1 (SHIP-1), which further inhibits the PI3K and MAPK signaling pathway [31]. Additionally, TIGIT can also indirectly improve the negative regulation function of DCs and Treg cells [36,37].

LAG-3 is not expressed by naive T-cells, but its expression is induced upon T-cell activation. Compared with PD-1, LAG-3 displays a moderate immunosuppressive activity. LAG-3 can transmit inhibitory signals via the KIEELE motif in its cytoplasmic tail, the FXXL motif in its membrane-proximal region (PR), or its C-terminal EX repeat [38]. However, the molecular mechanism by which LAG-3 inhibits T-cell activation is still largely unknown.

B and T lymphocyte attenuator (BTLA) is selectively expressed on Th1 cells and has been identified as an immune checkpoint receptor. Upon BTLA binding to herpesvirus entry mediator (HVEM), the BTLA cytoplasmic domains ITIM and ITSM bind to and activate the tyrosine phosphatases SHP-1 and SHP-2, which leads to the inhibition of lymphocyte-specific protein tyrosine kinase (LCK)-dependent T-cell activation [39,40]. Additionally, the third domain Grb-2-recognition motif in BTLA can recognize the Grb-2 protein, subsequently recruiting the PI3K protein subunit p85, stimulating the PI3K signaling pathway, and finally promoting cell proliferation and survival. Unlike other immune checkpoint regulators, BTLA can both positively and negatively co-stimulate T-cell regulation [41].

## 3. Immune Checkpoints in HIV

Human immunodeficiency virus (HIV) infects mainly T-cells. HIV infection destroys the host immune system and makes the infected individual increasingly more vulnerable to a range of infections, cancers, and other diseases. Recent research has shown that immune checkpoints extensively participate in HIV infection via their role in inhibiting T-cell function; the two main ways in which they act are by causing T-cell exhaustion and helping to establish HIV-latency reservoirs [42,43].

### 3.1. HIV-Specific T-cell Exhaustion Accompanies Immune Checkpoint Upregulation

T-cell exhaustion, which is a deterioration of T-cell function caused by exposure to persistent stimulation with high levels of antigen, is widely observed in chronic viral infection. It is defined by poor effector function, sustained inhibitory receptor expression, and a transcriptional state distinct from that of functional effector or memory T-cells [44]. Immune checkpoints are recognized inhibitory regulators of T-cell function and they have been reported to cause T-cell exhaustion. Upregulated expression of immune checkpoint proteins is a hallmark of T-cell exhaustion and dysfunction. During HIV infection, HIV-specific T-cells display upregulated expression of multiple immune checkpoint proteins [45], functional exhaustion, and failure to control viral infection (Table 2).

**Table 2:** Summary of the roles of immune checkpoints in viral infections.

Virus	Manifestations	Checkpoints Involved	Functions	Ref.
HIV	Establishment of HIV latency reservoirs	PD-1, CTLA-4, LAG-3, TIGIT	Participate in HIV infection and assist to virus escape from immune clearance	[43,46,47,48,49]
	CD4^+^ T-cell dysfunction	PD-1, CTLA-4, LAG-3, TIM-3	Cause many CD4 T-cells dysfunction and loss	[46,50,51,52,53]
	CD8^+^ T-cell exhaustion	PD-1, TIM-3, LAG-3, TIGIT	Lead to CD8^+^ T-cell functional impact such as decreased IL-2 secretion and T-cell proliferation	[42,46,54,55]
	Improved Treg proliferation	TIM-3, CTLA-4, PD-1	Decrease the HIV-specific immune responses, contributing to virus persistence	[56,57,58,59]
HBV	CD8^+^ T-cell exhaustion	PD-1, CTLA-4, Tim-3, TIGIT, LAG-3	Lead to CD8^+^ T-cell functional impact such as decreased IL-2 secretion and T-cell proliferation	[60,61,62,63]
	Upregulation on CD4^+^ T-cell	PD-1, CTLA-4, Tim-3	Increase Treg and regulate Th1/Th2 cytokine secretion	[64,65,66,67]
	Altered cytokine secretion	See Table 4		See Table 4
	Limited liver injury	LAG-3	Suppress T-cell function and mitigate liver injury	[68]
HCV	CD8^+^ T-cell exhaustion	PD-1, TIGIT, Tim3, CTLA-4	cause CD8^+^ T-cell functional defect such as decreased IL-2 secretion and T-cell proliferation	[69,70,71,72]
	Up-regulation on CD4^+^ T-cell	CTLA-4, TIM-3	Improve viral replication and persistence	[67,73]
	Altered cytokine secretion	See Table 4		See Table 4
Influenza	Decrease in CD8^+^ T-cell response	PD-1, Tim-3	Reduce the number of virus-specific CD8^+^ T-cell and cause T-cell dysfunction	[74,75,76,77,78,79,80,81,82]
	Up-regulation on both CD4^+^ and CD8^+^ T-cells from patients with influenza-associated encephalopathy	CTLA-4	Involved in influenza virus-associated encephalopathy	
SARS-CoV-2	Up-regulation on T-cells and NK cells from COVID-19 patients. Patient deteriorated from prodromal to symptomatic	PD-1, TIM-3	Mediate T-cell exhaustion and T-cell lymphopenia	[83,84,85]
HSV-1	CD8^+^ T-cell exhaustion	PD-1, LAG-3	Cause T-cell exhaustion and assist viral latency infection	[86,87,88]
	Reactivation of latency virus	PD-1, CTLA-4, TIM3	Mediate T-cell exhaustion and make them lost the control of spontaneous HSV-1 reactivation	[89]
EBV	Up-regulated expression	PD-1, CTLA-4, TIM3, 2B4	Provide T-cell dysfunction status to increase EBV escape and EBV latent infections	[90,91,92,93]
	Immune escape	PD-1		
Ebola virus	Upregulation on both CD4^+^ and CD8^+^ T-cells; Associated with high viremia and poor outcome	CTLA-4, PD-1	Mediate immune suppression	[94]

The exhaustion of CD8^+^ T-cells in HIV-infected individuals has been found to accompany the upregulated expression of various immune checkpoint proteins such as PD-1, TIM-3, LAG-3, and TIGIT [42,46,54,55,95]. Blockade of the PD-1/PD-L1 pathway significantly reduces HIV or SIV viral load and reinvigorates exhausted CD8^+^ T-cells in bone marrow-liver-thymus (BLT) humanized mice [96]. In addition, a blockade targeting TIM-3 and BTLA partially augmented the proliferation of HIV-specific CD8^+^ T-cells, whereas combined blockades of PD-1 and TIM-3 or BTLA enhanced the proliferation of CD8^+^ T-cells and the production of cytokines such as tumor necrosis factor (TNF)-α, interferon (IFN)-γ, and interleukin (IL)-13 [97].

As the HIV-infected target, HIV-specific CD4^+^ T-cells are severely suppressed during infection. Most activated CD4^+^ T-cells die from HIV cytopathogenicity or immune-mediated cell death. Compared with healthy controls, the expression levels of PD-1, CTLA-4, TIM-3, LAG-3, and TIGIT on effector CD4^+^ T-cells are elevated to varying degrees, accompanied by CD4^+^ T-cell function impairment and disease progression [47,50,51,52,98,99,100]. For example, upregulation of PD-1 and CTLA-4 expression is associated with higher plasma viral loads and lower CD4^+^ T-cell counts. Enhancements in CTLA-4, TIM-3, LAG-3, and TIGIT expression in HIV-infected CD4^+^ T-cells are also associated with the integration of HIV-DNA in these cells [46,50,51,53,101,102]. Additionally, the upregulation of CTLA-4 in CD4^+^ T-cells was reported to cause a high level of CCR5 expression on CD4^+^ T-cells, thereby supporting a vigorous HIV-1 infection [48], which suggests that CTLA-4 upregulation by HIV infection could increase CD4^+^ T-cell susceptibility to HIV infection.

Treg cells are a subset of CD4^+^ T-cells that express nuclear transcription factor fork head box P3 (Foxp3) and surface CD25. Under normal physiologic conditions, an increase in Treg cells protects tissues from damage due to excessive immune cell activation and proliferation [103]; however, it also plays a critical role in the immune suppressive reaction that is critical for chronic viral persistence [104,105]. Treg cells from HIV-1-infected patients express significantly higher levels of PD-1 [56,57], TIM-3 [106], CTLA-4 [107], and LAG-3 (Tr1) [58,59] compared with those from healthy individuals. Treg cells partly lose their ability to regulate T-cell function and proliferation during the chronic phase of HIV infection [108].

In summary, evidence suggests that the co-expression or single expression of immune checkpoint proteins is a key mechanism for the functional impairment of HIV-specific T-cells [109,110]. During acute HIV infection, progressive CD4^+^ T-cell dysfunction alters cytokine secretion [111] and reduces HIV clearance. Persistent TCR stimulation and the inflammatory environment during HIV infection spawn CD8^+^ T-cell exhaustion, resulting in more T-cell apoptosis and decreased T-cell cytotoxic ability. Therefore, untreated individuals eventually develop uncontrolled viremia and chronic HIV infection [112].

### 3.2. The Upregulation of Immune Checkpoint Proteins Helps to Establish a Latent HIV Reservoir

The progressive loss of CD4^+^ T-cells during HIV infection follows two patterns: the amount of HIV-infected CD4^+^ T-cells decreases sharply during acute infection [113,114] and the plasma CD4^+^ T-cell population is gradually drained further during chronic infection [115,116]. Some HIV-infected T-cells will survive immune clearance and become long-lived resting memory CD4^+^ cells, which harbor the replication-competent HIV provirus [117,118]. These cells are known as the functional HIV reservoir, which is the main cause of HIV chronic infection. Some studies have shown that HIV-DNA is often found in resting memory CD4^+^ T-cells [117]. The main cell types acting as HIV reservoirs include central memory (T_CM_) CD4^+^ T-cells, transitional memory (T_TM_) CD4^+^ T-cells, and specific differentiation subtypes, such as effector-memory (T_EM_) cells and terminally differentiated effector (TEMRA) cells [45,119].

Antiretroviral therapy (ART) can observably suppress viral replication, halt disease progression, and reduce viral loads in patients [120]. With such treatment, HIV infection can be manageable; however, if ART is stopped, the infection will rebound rapidly [121,122]. The post-integration latency of HIV is the biggest obstacle for treating HIV infection with ART because ART cannot eliminate non-replicating virus [123]. Until a new approach capable of eliminating the HIV reservoir is found, patients with HIV will require lifelong ART to ensure virus suppression.

Therapy targeting immune checkpoint proteins shows potential for eliminating HIV reservoirs. Multiple immune checkpoint receptors were found to be expressed on latent HIV-1 reservoir cells and associated with the amount of integrated HIV-DNA [102,124,125]. During ART, the CD4^+^ T-cells expressing PD-1, TIGIT, and LAG-3 alone or in combination were highly enriched for integrated viral genomes as compared with immune checkpoint receptor-negative CD4^+^ memory T-cells isolated from HIV-infected individuals [101]. The number of simultaneously co-expressed immune checkpoint receptors on CD4^+^ T-cells was closely related to the reservoir size and CD4^+^ T-cell counts [45,102,126]. These findings suggest that immune checkpoints might help to establish latent HIV reservoirs.

Even after years of ART, PD-1 is preferentially expressed on the surface of persistently infected cells, including HIV-1 latent reservoir cells such as T_CM_, T_EM_, and follicular helper T (Tfh) cells [49,126]. Recently, Tfh cells were found to serve as the major hideout for HIV replication and production in HIV-infected individuals undergoing ART [43,127,128]. The increased PD-1 expression by CD4^+^ T-cells is derived from the cell HIV-viral load. This expression is positively correlated with disease progression [42,125] and involved in the reduction of immune system activation that leads to the long-term survival of HIV latent reservoir T-cells [42,129]. In addition, CTLA-4^+^PD-1^−^ memory CD4^+^ T-cells have also been identified as HIV-DNA latency reservoir, and the major proportion of these cells were Treg cells [130,131].

In an in vitro HIV-latency model, a PD-L1/PD-1 blockade enhanced HIV-specific CD4^+^ T-cell proliferation and cytokine secretion [132]. Blocking PD-1 can potentiate viral reactivation from latency and reduce the size of HIV latency reservoirs [126,133,134]. One study found that blockade of a single immune checkpoint could retard but not eliminate the HIV latency reservoir because the exhausted T-cells could still express other immune checkpoint receptors. The combination of PD-1- and BTLA/TIM-3-specific antibodies showed enhanced therapeutic potential to augment T-cell responses [97]. Currently, several clinical trials aimed at determining the safety and efficacy of using ICIs as anti-HIV therapy are ongoing (Table 3).

## 4. Immune Checkpoints in Hepatitis Virus

Chronic infection caused by hepatitis B virus (HBV) or hepatitis C virus (HCV) is the main cause of liver cancer. Around 15%–40% of HBV-infected individuals are predisposed to cirrhosis or hepatocellular carcinoma (HCC) [135]. In Japan, HCV infection is the major risk factor for HCC (80%–90%) [136]. Three-quarters of HCV infections develop into chronic hepatitis. Fortunately, highly effective direct acting antivirals (DAA) targeting NS3 protease, NS5A polymerase, or NS5B polymerase can cure over 95% of HCV-infected individuals [137]. HBV is a small, enveloped, DNA virus. During HBV infection of hepatocytes, the relaxed circular DNA genome (rcDNA) of HBV is transported into the nucleus and converted into covalently closed circular DNA (cccDNA) to serve as a viral persistence reservoir [138], which is refractory to current antiviral therapies [139]. Life-long nucleoside/nucleotide analogue (NA) therapy can achieve long-term suppression of viral replication, but it cannot eliminate cccDNA. Consequently, the withdrawal of NA therapy poses the risk of viral rebound [140].

### 4.1. Hepatitis Virus Infection Upregulates Immune Checkpoint Proteins on T-cells

In acute HBV infection, immune checkpoints are upregulated in T-cells, but they mainly limit intrahepatic inflammation [141]. However, immune suppression due to immune checkpoints was also found to enhance viral persistence and lead to chronic infection. The expression levels of PD-1, TIM-3, TIGIT, LAG-3, and CTLA-4 have been observed to increase on peripheral HBV-specific CD8^+^ T-cells [60,61,62,68,142,143] (Table 2). This upregulation is related to T-cell exhaustion; for example, PD-1/PD-L1 ligation triggers T-cell apoptosis by various mechanisms, potentially assisting with viral persistence in the host [64,65,66].

The expression levels of PD-1, TIM-3, LAG-3, and CTLA-4 on CD4^+^ T-cells from HBV patients are significantly higher than those from healthy controls [64,65,66,67]. These levels are closely associated with a loss of cytokine production ability by CD4^+^ T-cells; this loss exacerbates CD8^+^ T-cell exhaustion, progressing HBV infection into the chronic phase. As a result of CD4^+^ T-cell dysfunction, CD8^+^ T-cells, particularly intrahepatic T-cells, exhibit progressive and gradual exhaustion during persistent viral infection, accompanied by persistent high viral loads and high antigen levels [143,144]. Similarly, in chronic HCV infection, the expression of PD-1, TIGIT, TIM-3 and CTLA-4 are also upregulated on CD8^+^ T-cells [69,70,71,72,145] and CD4^+^ T-cell [73,146]. It is associated with CD8^+^ T-cell exhaustion, CD4^+^ T-cell dysfunction and chronic low-level inflammation, which results in HCC development [145].

Treg cells play an important role in suppressing T-cell activation [147] and releasing regulatory cytokines such as IL-10, TGF-β, and IL-35 [148]. In the immune clearance and immune tolerance phases of HBV infection, the proliferation of Treg cells is promoted [149]. Levels of PD-1, CTLA-4, and TIM-3 expression are enhanced in Treg cells during HBV infection, and these proteins contribute to the regulatory functions of Treg cells [149,150,151,152].

PD-1 plays a critical role in T-cell exhaustion, especially in the persistent infection of hepatitis virus [153]. However, it was reported that T-cell exhaustion also occurred in PD-1 or PD-L1 knockout mice during chronic viral infection, accompanied by the upregulation of other immune checkpoint receptors [154]. These findings suggest that T-cell exhaustion is maintained by various immune checkpoints [68,143,155]. In isolated HBV-specific T-cells from a woodchuck model of HBV infection, blocking these immune checkpoints restored exhausted T-cells such that they to responded appropriately to stimulation by HBV antigens [63,156].

Importantly, treatment with an ICI must be monitored for adverse events, such as autoimmune events or increased liver inflammation. A recent study found that TIGIT blockade or deficiency in hepatitis B surface antigen (HBsAg) transgenic mice could promote the development of HCC by reversing the adaptive immune tolerance of hepatic CD8^+^ T-cells to HBsAg [60].

### 4.2. Immune Checkpoints Affect Chronic Hepatitis Virus Infection through Cytokine Regulation

Multiple studies with different models have shown that cytokines play a critical role in the control of HBV replication and chronic infection [157]. IFN-based therapy is the major treatment for chronic hepatitis B (CHB) [157,158,159,160]. Proinflammatory cytokines released by Th1 cells, such as IL-12, IL-2, IFN-γ, and TNF-α, can limit acute HBV infection and mediating non-cytolytic viral clearance via different signaling pathways [157]. In contrast, some cytokines like IL-10 and TGF-β contribute to impaired immune responses in chronic hepatitis [161,162].

During chronic hepatitis virus infection, immune checkpoints control T-cell cytokine production through various pathways (Table 3). The secretion of Th1 cytokines increases in the acute phase of hepatitis virus infection, then decreases rapidly with disease progression because of viral-specific T-cell dysfunction [163,164,165,166]. In contrast, Th2 cytokines, such as IL-4 and IL-10, are less affected, while increases in TGF-β and IL-35 levels can downregulate Th1 responses and decrease T-cell function [147,167,168,169]. Cytokine secretion varies among subsets of Th2 and Treg cells. Notably, a Th2 bias in the Th1/Th2 cytokine balance is not conducive to a successful antiviral immune response [73,170,171,172,173,174].

An immune checkpoint blockade or genetic knockdown can restore the production of proinflammatory cytokines (Table 3). Accumulating evidence shows that immune checkpoint blockades targeting PD-L1, LAG-3, CTLA-4, or TIM-3 improve the production of Th1 cytokines during HBV infection [63,175,176]. Although the detailed mechanism responsible for this effect is currently unclear, CTLA-4 and PD-1 were found to induce the differentiation of CD4^+^ Th cells toward Th2 cells and increase Th2 cytokine levels during HBV infection [73]. Additionally, PD-1 expression levels are significantly increased in HBV-specific CD4^+^ T-cells, lending support to the idea that immune checkpoints cause cytokine downregulation [143]. Because of its effect on restoring T-cell activity and enhancing cytokine production, immune checkpoint blockade holds promise as a useful antiviral immunotherapy; however, such therapy has the potential to induce the abnormal secretion of many cytokines and chemokines, known as a cytokine storm, or to trigger autoimmune diseases. Clinical trials are underway to study the efficacy of immunotherapy against infection with HBV or HCV (Table 4).

## 5. Immune Checkpoints in Influenza

Recent studies have revealed that immune checkpoints are also involved in influenza virus infection (Table 2). The expression of PD-L1 in human primary airway epithelial cells and the plasma level of Gal-9 were found to drastically increase in 24 h post-infection with influenza A virus (IAV) [74,75]. Furthermore, a PD-L1 blockade or knockout of Gal-9 in airway epithelial cells enhanced T-cell function and influenza virus clearance [74,76].

T-cell immunity is important for the clearance of respiratory viruses and control of the associated infection. However, T-cell effector functions are impaired in IAV infection because of the highly inflamed airway microenvironment. The expression levels of PD-1, TIM-3, LAG-3, and 2B4 on lung CD8^+^ T-cells during a primary influenza virus infection were significantly elevated, and these levels were even higher than the corresponding levels in individuals re-infected with this virus [77]. PD-1 ablation or blockade during IAV infection effectively increased IFN production and preserved CD8^+^ T-cell effector function, consequently decreasing viral titers [78,79]. Similarly, a significant elevation in TIM-3 expression was found in IAV-infected individuals. TIM-3/Gal-9 interaction was found to limit immune responses to influenza virus. Specifically, Gal-9 knockout mice were able to control IAV infection more successfully compared with wildtype mice, as supported by better virus-specific CD8^+^ T-cell responses and IAV-specific humoral responses [76]. Furthermore, enhanced CTLA-4 expression on CD8^+^ T-cells causes a downregulation of T-cell activation and is significantly associated with influenza encephalopathy [80].

Most influenza virus infections are self-limited because of the rapid clearance of virus. However, excessive immune responses to influenza virus can lead cytokine storms, which cause pathological damage to tissues. Immune checkpoints can limit such immune pathology; for example, TIGIT can reduce immune-mediated tissue damage in an IL-10-dependent manner during acute virus infection [81]. The negative regulatory function of T-cells was found to be improved in a mutant mouse with a deletion in the distal cytoplasmic domain of TIM-3. Furthermore, TIM-3 was reported to reduce pneumonitis in the setting of influenza infection by suppressing excessive T-cell activity [82].

In summary, immune checkpoints have been reported to play two opposing roles in influenza. Immune checkpoints can interfere with the clearance of influenza virus, but they can also limit the inflammatory immune response caused by influenza virus infection. Therefore, when using ICIs to treat acute influenza infection, the patients receiving this therapy must be closely monitored to avoid triggering an elevated lung inflammatory response. Two clinical studies regarding the safety of inactivated influenza vaccines in patients undergoing immunotherapy with ICIs have been completed (Table 4). The results show ICI immunotherapy did not increase the incidence or severity of immune responses to inactivated influenza virus. However, the response to active influenza virus in patients receiving ICI immunotherapy is still not clear.

## 6. Immune Checkpoints in COVID-19

SARS-CoV-2 is an emerging coronavirus that was identified as the causative pathogen for the respiratory illness COVID-19, which was first detected at the end of 2019. Inflammation-mediated damage is the main consequence of SARS-CoV-2 infection. Most severe COVID-19 cases exhibit a cytokine storm syndrome, leading to a poor prognosis [177]. Although the immune response during COVID-19 progression has been extensively studied in the short time since SARS-CoV-2 was identified [178,179,180], the role of immune checkpoints in this process is still not clear [181]. Reports indicate that the absolute numbers of peripheral blood NK cells, B-cells, and CD4^+^ and CD8^+^ T lymphocytes were all lower in patients with acute respiratory distress syndrome due to SARS-CoV-2 infection compared with those in healthy controls. Although fewer in number, these cells were hyperactivated, accompanied with an increase in serum levels of the proinflammatory cytokines IL-6, IL-10, and TNF-α [83,182]. Lymphopenia and the levels of IL-6, IL-10, and TNF-α were found to be negatively associated with COVID-19 patient survival [83,84]. PD-1 expression levels on NK cells and T-cells were found to be highly upregulated in COVID-19 patients [83,85,183]. Specially, an analysis on peripheral blood mononuclear cells from 14 patients in deteriorated condition from COVID-19 and 3 healthy controls revealed that COVID-19 patients have higher levels of both PD-1 and TIM-3 as well as remarkably elevated serum levels of IL-6, IL-10, and TNF-α. These findings suggest that immune checkpoint-mediated T-cell exhaustion is involved in the severe symptoms of COVID-19 [83].

Because immune checkpoints appear to be involved in the immune tolerance of SARS-CoV-2 infection, altering viral susceptibility through ICI therapy is theoretically possible. ICIs can usually restore the effector function of the CD8^+^ T-cells involved in defense against viral infections. However, the overlap between ICI mechanisms and COVID-19 pathogenesis raises concerns that ICI therapy may potentiate acute respiratory distress syndrome. Cancer immunotherapy as a risk factor has been examined in COVID-19 patients. When death was used as the examination endpoint, treatment with a PD-1 blockade was found not to be associated with an increased risk of COVID-19 severity in patients with cancer [184,185]. However, another ICI therapy study conducted on more patients used patient oxygen need as the examination endpoint and found that the disease severity in COVID-19 patients with cancer was associated with the ICI therapy; the authors concluded that ICI treatment was a predictor for hospitalization and severe outcomes [186]. In addition, autoimmune pneumonitis was observed in non-small cell lung cancer patients who were treated with ICI in up to 20% of cases [187]. Although ICIs are potentially beneficial immunomodulators of CD8^+^ T-cell-mediated immune surveillance, they can lead to different outcomes depending on the timing of this intervention [85]. Presently, the available data with which to clearly address the use of ICIs in patients with SARS-CoV-2 infection, with or without concurrent cancer, remain very limited.

## 7. Immune Checkpoints in Other Viruses

In addition to their functions in HIV, hepatitis virus, influenza virus, and SARS-CoV-2 infections, the roles of immune checkpoints in infections with other viruses are also being widely studied. The Epstein–Barr virus (EBV), initially described in 1964, was the first identified human tumor virus [188]; infection with this virus is linked to the development of lymphoma, nasopharyngeal carcinoma, and many types of malignancies. EBV can latently infect memory B-cells [189]. The expression levels of PD-1 and CTLA-4 are upregulated on EBV-infected T-cells, and PD-L1 is expressed on EBV-infected lymphoma cells [90,91]. A blockade of PD-1 and CTLA-4 enhanced T-cell responses and prolonged the survival time of EBV-infected humanized mice. However, another study found that PD-1^+^ CD8^+^ T-cells from EBV-infected mice with reconstituted human immune system components (huNSG mice) still retained proliferative capacity and that these animals had a worse outcome following treatment with anti-PD-1 antibodies [92]. The huNSG mice are NSG immunodeficient mice with HLA-A2 transgene and intrahepatically engraftment of HLA-A2^+^ CD34^+^ hematopoietic progenitor cells purified from human fetal liver tissue, which possess peripheral blood reconstitution of human CD45^+^ CD19^+^ B, CD3^+^ T, and NKp46^+^ NK cells, as well as CD4^+^ and CD8^+^ T-cells. The expression levels of TIM-3, LAG-3, BTLA-4, and 2B4 were found to be upregulated in EBV-specific T-cells and associated with high virus load. The upregulation of immune checkpoint receptors by T-cells indicates that these cells are dysfunctional, and a combination of T-cell dysfunction and PD-L1 overexpression contributes to the immune escape of both active and latent EBV [93].

Like EBV and HIV, herpes simplex virus (HSV) can cause a latent infection. HSV latent infection occurs in the trigeminal ganglion [190]. When HSV reactivates from latency, it can trigger more serious complications, such as herpes stromal keratitis (HSK) [191]. Related studies show that both CD4^+^ T-cells and CD8^+^ T-cells in draining lymph nodes have increased expression levels of PD-1, TIM-3, and LAG-3 after HSV infection [86,87]. This upregulated PD-1 and LAG3 expression led to the exhaustion of tissue-resident CD8^+^ T-cells and the loss of antiviral response to symptomatic reactivation, whereas a blockade of LAG-3 and PD-1 improved the function of HSV-specific CD8^+^ T-cells and significantly reduced the reactivation of latent HSV virus in a rabbit model [88,89].

Ebola virus is a single-stranded RNA virus belonging to the filovirus family. Infection with Ebola virus causes an acute, highly lethal hemorrhagic fever in humans. An analysis of the differences in T-cell activation status between fatalities and survivors of Ebola virus infection uncovered that CTLA-4 overexpression was correlated with high viremia [94].

## 8. Summary and Outlook

The immune responses to viral infection vary among viruses. In general, immune checkpoint-mediated immune suppression, an important mechanism for virus immune escape, is widely observed in viral infections. The upregulation of immune checkpoint proteins represents a critical point in the balance between an effective T-cell response and T-cell exhaustion. Shifting this balance through treatment with ICIs should restore T-cell competence and promote virus clearance. Thus, ICIs could be useful in the treatment of viral infections.

During some acute viral infections, such as those due to influenza virus or SARS-CoV-2, PD-1, and other immune checkpoint proteins are upregulated quickly to assist with viral immune escape and to counter the severe inflammatory response triggered by infection. Studies have recently uncovered roles played by immune checkpoints in chronic viral infections, like those due to HIV or HBV. The establishment of latent viral infection might largely rely on immune checkpoint-mediated immune suppression. Because viral infection, unlike most cancers, is usually accompanied by a strong inflammatory response, treating viral infections with ICIs poses a risk not present in their application to usual cancer treatment: restoring T-cell function might exacerbate the inflammatory response caused by the viral infection. Several questions remain regarding the application of such immunotherapy to viral infections. It will be important to determine: (1) which stage of infection is most appropriate for conducting an intervention with ICI immunotherapy; (2) how to accurately determine or predict the clinical responsiveness or adverse events from immune checkpoint blockade therapy; and (3) how to improve and control the clinical response rates of immune checkpoint blockade treatment. Therefore, further study and real-world observations are needed to confirm the potential utility of ICIs for antiviral therapy.

## Figures and Tables

**Table 1:** Immune checkpoints involved in viral infections and their T-cell inactivation pathways.

Immune Checkpoint	Super Family	Ligand	Expression	T-cell Inactivation Pathway	Ref.
PD-1	CD28	PD-L1/L2	T-cells, B-cells, natural killer T-cells, DCs and activated monocytes	(1) PD-1(ITSMs) → SHP2 → ZAP70/LCK → Ras-MEK-MAPK →AP1 (2) PD-1(ITSMs) → SHP2 → PI3K/AKT →NFAT	[20,21,22]
CTLA-4	CD28	B7 (CD80/CD86)	Active T-cells	CTLA-4 → PP2A → Akt → mTORc1 → regulates T-cell proliferation and differentiation	[25,26,27]
TIM-3	TIM	Galcetin-9	Th1, Tc1, Treg cells	(1) TIM-3 → release Bat3 → LCK / FYN → ZAP70 → Ras-MEK-MAPK → AP1 (2) TIM-3 → release Bat3 → LCK / FYN → ZAP70 → PLC-γ → NFAT	[32]
TIGIT	CD28	CD155 on DCs	T-cells and NK cells	TIGIT → SHIP1 → PI3K → AKT/PKC/IKK → NF-κB	[31,35]
LAG-3	Immunoglobulin	MHC class Ⅱ	Activated T-cells Treg cells, B-cells, DCs and NK cells	LAG- KIEELE motif /FXXL motif/ C terminus EX repeat → unknown	[38]
BTLA/CD160	CD28	HVEM	T-cells and B-cells, activated T-cells	(1) BTLA(ITIM/ITSM) → SHP-1/2 → LCK / FYN → ZAP70 → Ras-MEK-MAPK → AP1 (2) BTLA(ITIM/ITSM) → SHP-1/2 → LCK / FYN → ZAP70 → PLC-γ → NFAT	[39,41]

**Table 3:** Clinical trials on immune checkpoint inhibitors targeting viral infection.

Infection	Target	NCT ID	Research Title/Objective	Drug	Status
HIV	PD-1	NCT03239899	PD-1 Inhibition to Determine CNS Reservoir of HIV-Infection	Pembrolizumab	Phase Ⅰ
	PD-1	NCT03787095	Safety and Immunotherapeutic Activity of an Anti-PD-1 Antibody (Cemiplimab) in HIV-1-infected Participants on Suppressive cART	Cemiplimab	Phase Ⅰ
	IMC	NCT03354936	Assess the safety of the use of immune checkpoint inhibitors in HIV infected patients	Nivolumab, Pembrolizumab	Recruiting
	PD-1	NCT03367754	A Single Dose of Pembrolizumab in HIV-Infected People	Pembrolizumab	Phase Ⅰ
HBV	PD-L1	NCT03899428	Immune Checkpoint Therapy vs Target Therapy in Reducing Serum HBsAg Levels in Patients with HBsAg+ Advanced Stage HCC	Durvalumab	Phase Ⅱ
HCV	PD-1	NCT00703469	A Study of MDX-1106 to Treat Patients with Hepatitis C Infection (MDX1106-02)	MDX-1106 (PD-1 ab)	Phase Ⅰ
Influenza	PD-1	NCT03061955	Safety and Efficacy of Concurrent Administration of Influenza Vaccine in Patients Undergoing Anti-PD-1 Immunotherapy (Nivolumab, Pembrolizumab)	Nivolumab, Pembrolizumab	Completed
	PD-1/CTLA-4	NCT03590808	Influenza Vaccination in Patients Receiving Immune Checkpoint Inhibitor	Pembrolizumab, Nivolumab, Ipilimumab	Completed
HPV	CTLA-4	NCT01693783	Ipilimumab in Treating Patients with Metastatic or Recurrent Human Papilloma Virus-Related Cervical Cancer	Ipilimumab	Phase Ⅱ
EBV	PD-1	NCT03755440	PD-1 Antibody in EBV Positive Metastatic Gastric Cancer Patients	SHR-1210 (PD-1 ab)	Phase Ⅱ
	PD-1	NCT02488759	An Investigational Immuno-Therapy Study to Investigate the Safety and Effectiveness of Nivolumab, and Nivolumab Combination Therapy in Virus-associated Tumors (CheckMate358)	Nivolumab	Phase Ⅰ

**Table 4:** Cytokine secretion altered by immune checkpoints in chronic virus infection.

Chronic Virus	Cytokines Secretion during Viral Infection		Associated Immune Checkpoint	Effects	Ref.
HBV	TH-1/CD8^+^T	IFN-γ ↓	CTLA-4, PD-1, Tim-3, LAG-3, BTLA	Activate multiple immune responses Enhance Th1 responses Inhibit cccDNA replication and accumulation	[62,63,64,68,100,143,151,152,166]
		IL-2 ↓	CTLA-4, BTLA, PD-1, Tim-3, LAG-3	Induce differentiation and effector function of T-cell, NK and LAK	[62,64,67,143]
		TNF-α ↓	PD-1, Tim-3, LAG-3	Lead to direct clearance of virus-infection cells	[64,152,166]
		IL-12 ↓	PD-1	Impair host defense and viral clearance	[176]
	Treg	IL-10, TGF-β ↑	CTLA-4, PD-1, Tim-3	suppress effector T-cells and regulate liver fibrosis	[171,172,173]
	TH-2	IL-4, IL-5, IL-10 ↓	LAG-3, PD-1	Downregulate the activation of B- and T-cell proliferation Suppress TH1 response	[64]
	TH-17	IL-17 ↑	TIM-3	Promote the exacerbation of liver inflammation and sustain the proinflammatory response	[167]
HCV	TH-1/CD8^+^ T-cell	IFN-γ ↓	LAG-3, PD-1, Tim-3	Promote Th1 immune response, suppress Th2 and Th17 responses	[71,164]
		IL-2 ↓	PD-1, CTLA-4	Inactivate multiple immune-cell subsets, including T-cells, NK cells, B-cells, monocytes, macrophages, and neutrophils	[145]
		TNF-α ↓	LAG-3	Decrease the direct clearance of virus-infection cells	[164]
		IL-12 ↓	Tim-3	Decrease the stimulation of TH1 responses that are essential for host defense and pathogen clearance	[173]
	Treg	IL-10, TGF-β ↑	PD-1, Tim-3, CTLA-4	Limit the secretion of proinflammatory cytokines and suppress effector T-cells	[73,146,168]
	TH-2	IL-4, IL-5, IL-10 ↑	PD-1	Promote the activation of B and T-cell proliferation and limit the secretion of proinflammatory cytokines	[169]
	TH-17	IL-17 ↑	Tim-3	Promote the exacerbation of liver inflammation and injury	[173,174]
